# The Silent Revolution of the Genome: The Role of Optical Genome Mapping in Acute Lymphoblastic Leukemia

**DOI:** 10.3390/cancers17213445

**Published:** 2025-10-27

**Authors:** Claudia Simio, Matteo Molica, Laura De Fazio, Marco Rossi

**Affiliations:** Department of Hematology-Oncology, Azienda Universitaria Ospedaliera Renato Dulbecco, 88100 Catanzaro, Italy

**Keywords:** Philadelphia-like ALL, optical genome mapping, genomic profiling

## Abstract

**Simple Summary:**

Optical Genome Mapping (OGM) is an innovative technology that is transforming the genetic diagnosis of acute lymphoblastic leukemia (ALL). Thanks to its high resolution and direct DNA analysis, OGM enables the detection of complex rearrangements, deletions, and gene fusions that are often missed by conventional methods such as karyotyping, FISH, and PCR. In particular, it can identify clinically relevant alterations such as *IKZF1* deletions, *KMT2A* rearrangements, and kinase gene fusions involving *ABL1*, *PDGFRB*, *JAK2*, and *EPOR*, thereby improving molecular classification and guiding targeted therapy. The integration of OGM with next-generation sequencing (NGS) provides a comprehensive genomic overview, supporting more precise diagnosis, risk stratification, and personalized treatment in ALL.

**Abstract:**

**Background:** Acute lymphoblastic leukemia (ALL) is a genetically heterogeneous malignancy driven by structural variants (SVs) that impact diagnosis, prognosis, and treatment. Traditional methods such as karyotyping, FISH, and PCR often fail to detect cryptic or complex rearrangements, which are critical for accurate risk stratification. **Methods:** Optical Genome Mapping (OGM) is a technology that directly analyzes ultra-high-molecular-weight DNA, enabling the identification of balanced and unbalanced SVs, copy number variations (CNVs), and gene fusions with high resolution. This review compares the advantages and limitations of OGM versus standard techniques in ALL. **Results:** OGM improves ALL diagnosis by detecting clinically relevant alterations such as *IKZF1* deletions, cryptic *KMT2A* rearrangements, and kinase fusions, especially in cases with normal or uninformative karyotypes. It reduces artifacts by eliminating cell culture and shortens reporting times. OGM resolves complex events like intrachromosomal amplifications and chromothripsis, enhancing classification and therapy decisions. Limitations include reduced sensitivity in repetitive regions, challenges in detecting Robertsonian translocations, difficulties with complex ploidies, and lower sensitivity for low-frequency subclones. **Conclusions:** Integrating OGM with next-generation sequencing (NGS) allows comprehensive genomic profiling, improving diagnosis, prognosis, and personalized treatment in ALL. Future advancements promise to further enhance the clinical utility of OGM.

## 1. Introduction

Acute lymphoblastic leukemia (ALL) is a hematologic malignancy characterized by the clonal expansion of immature lymphoid precursors (lymphoblasts), which accumulate within the bone marrow and peripheral blood, thereby disrupting normal hematopoiesis. While ALL represents the most common leukemia subtype in pediatric populations, it also occurs in adults, with significant age-dependent differences in prognosis, treatment response, and underlying molecular features.

Over the past decades, molecular diagnostics have become integral to the clinical management of ALL. Comprehensive genomic and transcriptomic profiling has substantially advanced our biological understanding of the disease, leading to the identification of distinct molecular subtypes with well-defined prognostic and therapeutic implications.

Among high-risk genomic lesions, *IKZF1* deletions are particularly noteworthy, frequently observed in B-cell ALL and strongly associated with poor treatment response and increased relapse risk. Additionally, Philadelphia-like (Ph-like) ALL constitutes a high-risk subgroup defined by a gene expression profile reminiscent of *BCR::ABL1*-positive ALL, yet lacking the canonical translocation. This subset often harbors activating rearrangements involving kinase signaling pathways, which are potentially actionable with targeted therapies but portend a poor prognosis in the absence of such interventions [1]. Recent studies have uncovered a range of targetable gene fusions, such as those involving *ABL1* or *PDGFRB*, which may respond to tyrosine kinase inhibitors, and rearrangements of *JAK2*, *EPOR*, or *IL7R*, which confer sensitivity to JAK inhibitors. These findings have expanded the landscape of precision medicine in ALL, offering new avenues for risk-adapted therapy.

Additional adverse prognostic markers include hypodiploidy—particularly cases with fewer than 44 chromosomes—associated with highly aggressive clinical behavior, and *KMT2A* rearrangements, which are linked to primary chemoresistance and early relapse. Co-occurring deletions in lymphoid transcriptional regulators, such as *PAX5*, *EBF1*, and *ETV6*, may further worsen prognosis when present alongside IKZF1 deletions, delineating the “*IKZF1plus*” phenotype, a distinct high-risk entity. These genomic alterations are now central to risk stratification models and are critical in guiding personalized therapeutic approaches, including the early incorporation of allogeneic hematopoietic stem cell transplantation or molecularly targeted agents in high-risk settings.

Currently, standard genomic assessment relies on conventional cytogenetics, fluorescence in situ hybridization (FISH), polymerase chain reaction (PCR), and increasingly, next-generation sequencing (NGS). While these methods have enabled significant advances, they remain limited by variable resolution, inability to detect cryptic or complex structural variants, and lengthy turnaround times [2].

In this context, Optical Genome Mapping (OGM) has emerged as a powerful, high-resolution platform for structural variant detection. By enabling direct visualization of ultra-long DNA molecules, OGM can detect a wide spectrum of genomic aberrations—including translocations, inversions, deletions, duplications, and gene fusions—many of which may be cryptic or complex and elude detection by conventional methodologies such as karyotyping, FISH, or PCR. Due to its unbiased, genome-wide detection capabilities, OGM is proving particularly valuable in the comprehensive molecular characterization of ALL, with potential implications for both diagnostics and prognostics, as well as for the rational selection of targeted therapeutic strategies [3].

## 2. Optical Genome Mapping: Mechanism of Action, Clinical Utility, and Applications in ALL

Optical Genome Mapping represents a state-of-the-art platform for high-resolution, genome-wide detection of structural variants (SVs). By enabling the direct visualization of ultra-long DNA molecules, OGM facilitates the comprehensive identification of a broad spectrum of structural alterations—including translocations, inversions, deletions, duplications, amplifications, and gene fusions—even when such aberrations are cryptic, complex, or unbalanced, and thus remain undetectable by conventional cytogenetic approaches [4].

### 2.1. Mechanism of Action

Optical Genome Mapping (OGM) detects structural variants by imaging ultra-high-molecular-weight (UHMW) DNA (>150 kb). Intact genomic DNA is extracted without fragmentation, enzymatically labeled at specific recognition sites, linearized in microfluidic nanochannels, and imaged through high-resolution fluorescence microscopy. The resulting optical maps are computationally aligned to a reference genome to identify structural anomalies based on deviations in label positioning. The Saphyr system (Bionano Genomics) is currently the most widely used platform, enabling high-throughput acquisition of millions of DNA molecules and fully automated analysis via proprietary bioinformatics pipelines [4].

### 2.2. Clinical Applications

Optical Genome Mapping is currently being integrated into clinical practice and has demonstrated particular utility at multiple stages of the diagnostic and therapeutic workflow:

- Initial diagnosis for comprehensive structural genomic characterization, especially in cases with uninformative karyotypes or suspected cryptic abnormalities;
- Prognostic stratification through the identification of high-risk alterations that remain undetectable by conventional methods;
- Definition of targetable alterations such as gene fusions involving *ABL1*, *PDGFRB*, *JAK2*, and others;
- Detection of complex anomalies, particularly in cases with complex karyotypes or uninterpretable genomic alterations;
- Follow-up and relapse monitoring (experimental) to track the emergence of novel genomic changes at relapse, although this is not yet an established routine clinical application;

Currently, OGM is not recommended for minimal residual disease (MRD) monitoring or routine follow-up testing during remission due to its relatively limited limit of detection (LOD)—approximately 5% for structural variants and 10–15% for copy number variants [5].

### 2.3. Application in ALL

In acute lymphoblastic leukemia (ALL), OGM contributes to the comprehensive genomic characterization of the disease. It has proven particularly useful in cases where conventional cytogenetic analysis is unsuccessful or yields normal results, allowing clarification of discordant molecular and clinical findings. OGM also facilitates the discovery of novel gene fusions and complex rearrangements relevant to disease classification and potential therapeutic targeting.

Emerging evidence supports its exploratory use for tracking clonal genomic evolution at relapse, although its role in longitudinal monitoring remains under investigation [6].

**Figure 1:**
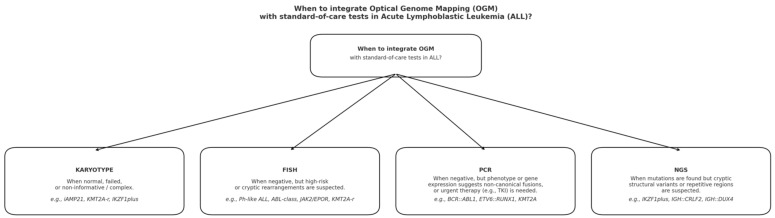
Diagnostic workflow for integration of Optical Genome Mapping (OGM) with standard-of-care tests in acute lymphoblastic leukemia (ALL). OGM should be incorporated when conventional assays yield normal, failed, or inconclusive results, or when clinical and molecular findings are discordant. Specifically, OGM complements karyotyping in cases with uninformative or complex profiles (e.g., i*AMP21*, *KMT2A-r*, *IKZF1plus*), FISH when cryptic or high-risk rearrangements are suspected (e.g., *Ph-like ALL*, *ABL-class*, *JAK2/EPOR*, *KMT2A-r*), PCR when canonical fusions are negative but phenotypic clues suggest hidden lesions (e.g., *BCR::ABL1*, *ETV6::RUNX1*, *KMT2A*), and NGS when sequence-based tests reveal mutations but structural abnormalities remain unresolved (e.g., *IKZF1plus*, *IGH::CRLF2*, *IGH::DUX4*).

To illustrate the diagnostic integration of Optical Genome Mapping (OGM) within the current standard-of-care workflow for acute lymphoblastic leukemia (ALL), Figure 1 presents a schematic summary of the specific contexts in which OGM should complement conventional assays such as karyotyping, FISH, PCR, and NGS.

This integrated workflow highlights how OGM complements existing methodologies, improving detection of clinically relevant cryptic rearrangements and supporting comprehensive genomic profiling in ALL.

## 3. Advantages of Optical Genome Mapping in ALL

OGM offers a unified, high-resolution approach to structural variant detection, integrating multiple cytogenetic and molecular assays into a single workflow. As summarized in Table 1, it enables detailed breakpoint mapping, identification of cryptic rearrangements, and precise delineation of copy number changes—all of which refine risk stratification and inform therapeutic decision-making.

By complementing next-generation sequencing (NGS), which excels at detecting small sequence-level variants, OGM provides a genome-wide structural perspective essential for a complete molecular diagnosis in ALL.

### 3.1. Comprehensive Diagnostic Approach: Simultaneous Detection of SVs, CNVs, Deletions, Translocations, and Gene Fusions in a Single Assay, Streamlining Diagnostic Workflows

Optical Genome Mapping enables the simultaneous detection of structural variants, copy number variations (CNVs), and both balanced and unbalanced translocations within a single comprehensive assay, thereby obviating the need for a fragmented, multimodal diagnostic approach traditionally reliant on conventional karyotyping (FISH), MLPA, array-CGH, or RT-PCR. Each of these conventional modalities is constrained by intrinsic limitations that may impede thorough genomic profiling. Conventional cytogenetics, for example, is limited to the analysis of a small number of metaphases (typically ~20) and requires actively dividing cells, rendering it ineffective in hypocellular or non-proliferative samples. While FISH offers superior resolution compared to karyotyping, its scope is restricted to targeted loci dictated by probe design, limiting the detection to known or suspected aberrations. Moreover, breakpoint resolution is often coarse, and certain rearrangements may evade detection altogether.

A cardinal advantage of OGM resides in its capacity to concurrently detect both balanced and unbalanced chromosomal rearrangements with markedly enhanced breakpoint precision relative to conventional cytogenetics and FISH. This technology facilitates genome-wide mapping of balanced translocations at a resolution unattainable by standard methodologies, particularly in instances where rearrangements are cryptic or cytogenetically silent, which conventional approaches and array-based platforms frequently fail to accurately localize or identify. A paradigmatic example is the balanced translocation involving *UBE3C* and *MSI2*, implicated in the pathogenesis of acute lymphoblastic leukemia (ALL) and aggressive acute myeloid leukemia (AML) subtypes, which can be precisely delineated by OGM [7].

Although next-generation sequencing (NGS) remains the gold standard for nucleotide-level characterization of somatic mutations—including single-nucleotide variants (SNVs), small insertions and deletions (indels), and copy number variations (CNVs)—its routine application is predominantly limited to targeted gene panels. Furthermore, NGS [8] exhibits inherent limitations in detecting complex structural variants (SVs) and alterations within repetitive or low-complexity genomic regions. In this setting, Optical Genome Mapping (OGM) does not supplant NGS but rather complements it, as OGM lacks sensitivity for single-nucleotide changes and small indels. The combined use of these modalities therefore enables a more comprehensive genomic landscape characterization, with substantial implications for diagnosis, prognosis, and therapeutic stratification.

While single-nucleotide polymorphism (SNP) arrays are widely regarded as the benchmark for CNV detection, many array-identified alterations correspond to translocation breakpoints that OGM can resolve with markedly greater precision. Notably, OGM leverages ultra-high-molecular-weight DNA molecules and de novo assembly algorithms to precisely delineate the regions involved in unbalanced translocations, achieving breakpoint resolution on the order of kilobases. This enhanced resolution permits unequivocal discrimination between CNVs and SVs, improving interpretative accuracy even in samples with low tumor cell content or low variant allele frequencies, contexts in which array-based platforms often underperform.

Importantly, OGM is capable of detecting insertions and deletions as small as ≥500 base pairs and localizing translocation breakpoints with high fidelity. This superior resolution relative to conventional cytogenetics, FISH, and array technologies stems from OGM’s capacity to analyze DNA molecules spanning from several hundred base pairs up to one megabase. Compared to short- and long-read sequencing approaches, optical genome mapping provides a more faithful and granular representation of structural variants, proving especially adept at resolving complex or cryptic rearrangements that evade detection by sequencing alone [9].

### 3.2. No Requirement for Cell Culture or DNA Amplification

A key advantage of Optical Genome Mapping (OGM) is its minimal input requirement, making it particularly well-suited for the analysis of paucicellular hematologic samples. High-resolution genome mapping can be achieved with as few as 1.5 million cells—substantially less than the ≥10 million cells typically required for conventional karyotyping or the 2–5 million cells needed for RT-PCR.

In contrast to karyotyping and FISH, OGM does not rely on ex vivo cell proliferation, thereby eliminating the need for mitotic cell culture. This bypasses one of the major limitations of classical cytogenetics: culture failure in samples with low mitotic activity or compromised cell viability. The absence of a cell culture step also enables successful analysis of cryopreserved or fixed samples, which are frequently unsuitable for metaphase-based techniques. As a result, OGM expands the range of clinically usable specimens and improves diagnostic access in challenging or suboptimal conditions.

At the molecular level, OGM offers a distinct advantage over sequencing-based approaches in that it does not require PCR amplification. This eliminates amplification bias and preserves the native architecture of the genome, enabling more accurate quantification of structural variants and a more faithful assessment of allele frequencies.

Moreover, OGM provides superior genomic coverage compared to whole-genome sequencing (WGS), achieving an effective depth of approximately 200–300×, as opposed to the ~60× typically achieved by WGS. This increased coverage enhances analytical sensitivity, allowing for the detection of low-frequency structural variants with a limit of detection around 5%. Such sensitivity is particularly valuable in the context of hematologic malignancies, where minor or subclonal populations may carry clinically actionable alterations or drive disease progression [10].

### 3.3. High Sensitivity and Resolution

Optical Genome Mapping (OGM) offers unparalleled sensitivity and resolution in the structural interrogation of hematologic malignancies. In cases of complex or ambiguous karyotypes, OGM enables precise delineation of cytogenomic architecture, outperforming conventional cytogenetic techniques in the identification of clinically relevant chromosomal abnormalities. By uncovering cryptic or compound rearrangements with higher fidelity, OGM enhances risk stratification and contributes to a more refined prognostic assessment—bridging the gap between cytogenetics and precision medicine.

#### 3.3.1. High-Resolution CNV Detection: The Advantage of OGM in Identifying Submicroscopic Deletions

Optical Genome Mapping (OGM) offers exceptional sensitivity for the detection of copy number variations (CNVs), with a resolution sufficient to identify submicroscopic deletions that are often undetectable by conventional genomic platforms. A compelling example is the identification of a 2.3 kb deletion involving the *SETD2* gene, which was not detected by standard methodologies such as SNP arrays, fluorescence in situ hybridization (FISH), or conventional karyotyping. This case underscores the superior resolving power of OGM in mapping clinically significant genomic regions with high precision, particularly in contexts where traditional cytogenetic approaches fail to capture cryptic or low-level structural aberrations. The ability to resolve such minute alterations expands the diagnostic reach of genome profiling in acute lymphoblastic leukemia and other hematologic malignancies [11].

#### 3.3.2. Detection and Structural Characterization of Recurrent Chromosomal Rearrangements

The identification of recurrent chromosomal rearrangements represents a pivotal factor in risk stratification and therapeutic decision-making in acute lymphoblastic leukemia. These abnormalities are typically detected by FISH; however, OGM has demonstrated the ability not only to detect such gene fusions with higher sensitivity—often revealing a greater variant allele frequency (VAF)—but also to provide a more detailed characterization of their structural complexity. A representative example is the resolution of a complex balanced translocation, undetected by conventional techniques, identified as a three-way rearrangement t(2;12;21)(p22.1;p13.2;q22.12), involving *ETV6*, *RUNX1*, and a third genomic region. OGM enabled precise breakpoint mapping, contributing to a deeper understanding of the underlying architecture of the fusion event [12].

#### 3.3.3. Identification of Prognostically Adverse Submicroscopic Deletions

Optical Genome Mapping (OGM) enables high-resolution detection of structural genomic alterations with established prognostic significance in acute lymphoblastic leukemia (ALL), including deletions affecting *CDKN2A/B*, *IKZF1*, and *PAX5* loci. Among these, *CDKN2A/B* loss has been consistently associated with a more aggressive clinical course. Recent evidence demonstrates that patients harboring *CDKN2A/B* deletions experience significantly reduced disease-free survival (DFS) and overall survival (OS) compared to their wild-type counterparts, highlighting the adverse prognostic impact of this lesion. Owing to its ability to detect such deletions—even when submicroscopic or embedded within complex genomic rearrangements—OGM emerges as a powerful tool for refined risk stratification and the implementation of precision-tailored therapeutic strategies in ALL [13].

A recently characterized high-risk subgroup in acute lymphoblastic leukemia (ALL) is defined by the *IKZF1plus* profile, which is marked by the co-occurrence of *IKZF1* deletions alongside deletions in *CDKN2A/B*, *PAX5*, or the *PAR1* region, in the absence of *ERG* gene alterations. Patients harboring this genomic configuration exhibit significantly reduced event-free survival (EFS) and higher relapse rates, closely correlated with levels of measurable residual disease (MRD). Although these abnormalities can be detected by SNP-array, Optical Genome Mapping (OGM) offers distinct advantages in cases involving highly repetitive genomic regions, such as those affecting *IKZF1*, where probe density in array-based platforms is often insufficient for adequate resolution. By providing high-resolution, probe-independent detection of structural variants, OGM enables more precise mapping of these lesions, enhancing risk stratification and improving prognostic assessment in ALL [14].

#### 3.3.4. High-Resolution Detection of i*AMP21* and Chromothripsis of Chromosome 21

Intrachromosomal amplification of chromosome 21 (i*AMP21*) defines a distinct high-risk molecular subtype of pediatric B-cell acute lymphoblastic leukemia (B-ALL), characterized by complex structural rearrangements including focal amplifications, inversions, and multiple deletions within chromosome 21. Although traditionally identified by fluorescence in situ hybridization (FISH), this approach offers limited resolution and may fail to fully characterize the underlying genomic architecture. Optical Genome Mapping (OGM) enables high-resolution, genome-wide detection of i*AMP21*, while also uncovering evidence of chromosome 21 chromothripsis—an extreme form of genomic fragmentation and rearrangement. The presence of i*AMP21* and associated chromothripsis has been strongly correlated with poor outcomes under standard therapy protocols, underscoring the clinical utility of OGM in refining risk stratification and guiding therapeutic decision-making in pediatric B-ALL [15].

#### 3.3.5. High-Resolution Detection of Chromosomal Breakpoints

High-resolution breakpoint mapping by Optical Genome Mapping enables precise detection of clinically actionable gene rearrangements in acute lymphoblastic leukemia (ALL), including *MEF2D::CSF1R* and *PAX5::JAK2* fusions—hallmarks of the Philadelphia-like (Ph-like) ALL subtype. *MEF2D*-rearranged leukemic cells have demonstrated pronounced in vitro sensitivity to histone deacetylase inhibitors (HDACi), likely driven by overexpression of *HDAC9*, a direct transcriptional target of *MEF2D* fusion proteins. These observations support the rationale for incorporating HDAC inhibitors into targeted therapeutic strategies aimed at improving clinical outcomes in this high-risk molecular subgroup [16].

### 3.4. Uncovering Cryptic and Novel Rearrangements

Optical Genome Mapping (OGM) enables the high-resolution detection of both balanced and unbalanced chromosomal rearrangements (Table 2), offering precise delineation of genomic breakpoints. This advanced level of structural insight not only facilitates the comprehensive characterization of established aberrations but also supports the discovery of previously unrecognized or cryptic variants with potential diagnostic, prognostic, or therapeutic significance in acute lymphoblastic leukemia.

#### 3.4.1. Precise Identification of the *ETV6::ABL1* Fusion

Acute lymphoblastic leukemia harboring the *ETV6::ABL1* fusion constitutes a rare but clinically relevant subtype, typically associated with poor prognosis. Importantly, this rearrangement confers sensitivity to tyrosine kinase inhibitors (TKIs), underscoring the need for prompt and accurate identification. However, the cryptic nature of the fusion—often resulting from complex or unbalanced structural rearrangements—can render it undetectable by conventional FISH. OGM offers a distinct advantage in this context, enabling high-resolution, genome-wide detection of structural variants by directly mapping breakpoint regions. This capability allows for the reliable identification of *ETV6::ABL1* fusions, even when masked by structural complexity, and supports more precise risk stratification and therapeutic decision-making [17].

#### 3.4.2. Identification of a Novel *WDFY2::ARID2* Gene Fusion

Optical Genome Mapping (OGM) enabled the precise identification of a t(12;13) chromosomal translocation generating a novel *WDFY2::ARID2* gene fusion. *ARID2*, a critical subunit of the SWI/SNF chromatin remodeling complex, is well-established for its tumor suppressor functions and essential role in regulating hematopoietic stem cell differentiation. Importantly, this translocation is frequently accompanied by an intragenic deletion within *ARID2*, likely resulting in functional haploinsufficiency.

Moreover, recent evidence implicates ARID family members as potential predictive biomarkers for response to immune checkpoint blockade therapies, underscoring the clinical and therapeutic significance of these genomic alterations [18].

#### 3.4.3. Identification of the *FLI1::EWSR1* Translocation

The *FLI1::EWSR1* gene fusion is a rare and atypical event in acute lymphoblastic leukemia (ALL), not typically classified among the canonical translocations driving the disease. Despite its rarity, emerging evidence suggests that this fusion may have profound clinical relevance. Leukemias harboring such complex or infrequent rearrangements frequently correlate with adverse clinical outcomes and reduced responsiveness to standard therapeutic regimens, including chemotherapy and immunotherapy. Mechanistically, the *FLI1::EWSR1* fusion is hypothesized to contribute to leukemogenesis by disrupting critical regulatory pathways governing cell cycle progression and proliferation, thus facilitating the clonal expansion of malignant lymphoblasts and promoting a more aggressive disease phenotype [19].

#### 3.4.4. Characterization of a Novel *TMEM272::KDM4B* Fusion Resulting from t(13;19)(q14.13;q13.3)

The t(13;19)(q14.13;q13.3) translocation leads to the formation of a novel *TMEM272::KDM4B* fusion gene in acute lymphoblastic leukemia (ALL). The oncogenic potential and functional consequences of this fusion remain to be elucidated. *KDM4B* is frequently overexpressed across a broad spectrum of solid tumors, including breast, colorectal, ovarian, lung, gastric, and prostate cancers. Its overexpression correlates with demethylation of the epigenetic mark H3K9me3, resulting in aberrant gene expression and genomic instability that promote tumorigenesis and cancer progression. Despite this, the role of *KDM4B* in hematologic malignancies such as ALL is not yet well understood and requires further comprehensive investigation. Additionally, the biological and clinical significance of the *TMEM272::KDM4B* fusion warrants validation in larger patient cohorts to clarify its relevance in leukemogenesis and potential as a therapeutic target [15].

#### 3.4.5. Characterization of the Novel *LMNB1::PPP2R2B* Fusion

The novel *LMNB1::PPP2R2B* gene fusion, involving LMNB1—which encodes lamin B1, a key component of the nuclear lamina—and PPP2R2B, a regulatory subunit of protein phosphatase 2A (PP2A), represents a previously unreported chromosomal rearrangement with potential implications in leukemogenesis. This fusion may disrupt critical cellular pathways, including cell proliferation and apoptosis regulation, thereby contributing to leukemic transformation. In our cohort, the majority of cases harboring the *LMNB1::PPP2R2B* fusion also carried the canonical *ETV6::RUNX1* fusion, suggesting a cooperative role in disease pathogenesis. These findings implicate *LMNB1::PPP2R2B* as a possible modulator of tumor biology specifically within the *ETV6::RUNX1*-positive acute lymphoblastic leukemia (ALL) subtype, warranting further functional studies to elucidate its mechanistic impact and potential as a therapeutic target [15].

#### 3.4.6. Characterization of the *JAK2::NPAT* Fusion

JAK2 (Janus kinase 2) is a non-receptor tyrosine kinase essential for transducing cytokine signals that regulate hematopoietic cell proliferation, differentiation, and survival. NPAT (nucleolar protein, activated by T antigen) is a nuclear protein implicated in cell cycle progression, particularly during the G1/S transition. Recurrent *JAK2* fusion events involving diverse partner genes—including *ATF7IP*, *EBF1*, *PAX5*, *SSBP2*, *RNPC3*, *GOLGA5*, and *PCM1*—have been described in a subset of acute lymphoblastic leukemia cases. These rearrangements result in constitutive JAK2 activation, driving leukemogenesis through cytokine-independent signaling. Importantly, JAK2-driven oncogenic signaling is amenable to pharmacologic inhibition with selective JAK inhibitors, such as ruxolitinib, underscoring a potential therapeutic avenue for this molecularly defined subset of ALL [11].

#### 3.4.7. Characterization of the Novel *OSBPL3::NRIP1* Fusion

OSBPL3 (Oxysterol-binding protein-like 3) encodes a protein primarily implicated in bone homeostasis, though emerging evidence suggests it may also participate in broader, yet incompletely defined, cellular processes. Gene fusions involving OSBPL3 may disrupt regulatory pathways controlling cell proliferation and the cell cycle, thereby contributing to malignant transformation.

NRIP1 (Nuclear Receptor Interacting Protein 1) functions as a transcriptional coregulator through its interaction with nuclear receptors, which play a central role in the control of cell growth, differentiation, and cycle progression. Disruption of NRIP1 function through gene fusion events may lead to aberrant transcriptional programs and promote oncogenic behavior.

A complex translocation, t(4;7;21), resulting in the novel *OSBPL3::NRIP1* fusion, was recently identified via (OGM). However, due to the rarity of this event, clinical and prognostic data remain insufficient to assess its pathogenic significance or potential impact on disease trajectory. Further functional and clinical studies are needed to define the biological relevance and therapeutic implications of this genomic alteration [15].

#### 3.4.8. Characterization of the Translocation t(5;12;21)

The pathogenic significance of the complex translocation t(5;12;21)(q11.2;p11.23;q22.12) remains incompletely understood. However, isolated t(5;12) rearrangements have been reported in patients with chronic eosinophilic leukemia (CEL), some of whom achieved clinical responses with dasatinib [20], suggesting sensitivity to tyrosine kinase inhibition. Moreover, Dun et al. described a case of acute lymphoblastic leukemia harboring the t(5;12;21) translocation, in which a *BCR::ABL1*-positive subclone emerged during disease progression [21].

These observations highlight the potential for targetable clonal evolution in the context of complex chromosomal rearrangements. Accordingly, longitudinal molecular surveillance is essential to detect emerging driver lesions that may inform the use of tyrosine kinase inhibitors (TKIs) and other precision therapies.

#### 3.4.9. Characterization of the Novel *RUNX1::SPG7* Fusion

RUNX1 is a transcription factor critical for normal hematopoietic development and is frequently involved in oncogenic fusion events in leukemia, most notably in acute myeloid leukemia with the t(8;21) translocation and in acute lymphoblastic leukemia harboring the *ETV6::RUNX1* fusion. In contrast, *SPG7*, a gene primarily implicated in maintaining mitochondrial function, has not previously been associated with oncogenic fusions in hematologic malignancies.

At present, clinical and functional data are insufficient to determine the prognostic or therapeutic significance of such fusion events involving *SPG7*, and their biological relevance remains to be established [15].

### 3.5. Reduction in Diagnostic Time and Complexity

The conventional diagnostic pathway for leukemia relies on a tiered, multi-assay strategy in which each test is selected based on the results of the preceding one. G-banded karyotyping, while standard for detecting large chromosomal aberrations (>5 Mb), offers limited resolution and often requires follow-up with targeted assays. FISH is typically employed to identify specific, clinically suspected rearrangements such as *BCR::ABL1* or *ETV6::RUNX1*, whereas RT-PCR or NGS are necessary for detecting cryptic fusions and sequence-level alterations. Additional platforms, such as array-CGH or SNP arrays, are required to identify submicroscopic deletions and duplications.

This fragmented approach can extend diagnostic turnaround times to 10–20 working days or more, and may fail to capture relevant genomic lesions not explicitly tested for. OGM offers a comprehensive, single-platform alternative capable of detecting large structural variants, copy number changes, cryptic fusions, and complex chromosomal rearrangements in a single assay. This technology significantly reduces diagnostic complexity and shortens turnaround times to 4–7 working days, even in laboratories with limited prior expertise. By consolidating multiple assays into one, OGM minimizes the need for sequential testing, repeat analyses, or assay-specific sample requirements. Although initial setup costs may be higher, per-sample cost reductions of up to 50% have been reported when compared with conventional multi-platform workflows. 

From an economic and healthcare perspective, the reported per-sample cost reduction of up to 50% primarily reflects the consolidation of multiple sequential tests—such as karyotyping, targeted FISH panels, SNP-array, and RT-PCR—into a single OGM workflow. This integration streamlines laboratory operations by reducing reagent consumption, hands-on time, and the need for repeated analyses, thereby decreasing overall diagnostic costs. Moreover, shorter turnaround times may yield indirect economic benefits, enabling earlier initiation of targeted therapies (e.g., tyrosine kinase inhibitors or transplant procedures) and potentially reducing hospital stays and the need for additional testing.

Although the magnitude of cost savings depends on factors such as case volume, platform utilization, and the need for confirmatory assays, the overall impact supports the economic sustainability of integrating OGM into precision diagnostics for acute lymphoblastic leukemia [22]

This paradigm shift not only improves diagnostic efficiency and comprehensiveness but also enhances the economic sustainability of precision diagnostics in hematologic malignancies [23].

## 4. Limitations and Drawbacks of Optical Genome Mapping in ALL

Compared to conventional diagnostic modalities, Optical Genome Mapping (OGM) exhibits inherent limitations (Table 3) in the context of acute lymphoblastic leukemia (ALL). Although OGM enables comprehensive detection of structural variants across the genome, it is inherently constrained in identifying balanced chromosomal rearrangements within highly repetitive genomic regions, such as centromeres and the short arms of acrocentric chromosomes, where conventional cytogenetic techniques like G-banded karyotyping and fluorescence in situ hybridization (FISH) retain superior sensitivity. Furthermore, OGM demonstrates reduced sensitivity in detecting structural variants present at low variant allele fractions, limiting its capacity to identify subclonal populations, a limitation effectively addressed by molecular assays, including RT-PCR and next-generation sequencing (NGS). Consequently, despite its high-resolution and broad-spectrum capabilities, OGM necessitates complementary use alongside established cytogenetic and molecular approaches to achieve a comprehensive and clinically actionable genomic profile in ALL.

### 4.1. Challenges of Optical Genome Mapping in Detecting Balanced Rearrangements and Low Allele Frequency Variants

Optical Genome Mapping (OGM) demonstrates intrinsic limitations in resolving balanced chromosomal rearrangements occurring within highly repetitive genomic regions, such as centromeres and the short arms of acrocentric chromosomes. Its sensitivity is also reduced for structural variants present in subclonal populations with low variant allele frequencies, potentially hindering the detection of clinically relevant minor clones [24].

### 4.2. Limitations of OGM in Identifying rob(15;21) in iAMP21 ALL

Optical Genome Mapping (OGM) lacks the capability to detect Robertsonian translocations, such as rob(15;21)(q10;q10)c, which constitute a known predisposing factor for acute lymphoblastic leukemia (ALL) with intrachromosomal amplification of chromosome 21 (i*AMP21* ALL). Due to the absence of unique labeling sites in the pericentromeric regions involved in these fusions, such rearrangements remain undetectable by OGM, highlighting a critical diagnostic blind spot in cases where Robertsonian translocations have pathogenic relevance [25].

### 4.3. Challenges in Interpreting Complex Ploidy States with Optical Genome Mapping

Accurate discrimination among hypodiploidy, euploidy, and hyperdiploidy is essential for the diagnostic classification and risk stratification of B-cell acute lymphoblastic leukemia. While Optical Genome Mapping enables high-resolution detection of structural genomic abnormalities using ultra-high-molecular-weight DNA, it does not provide a direct quantification of whole-chromosome copy number, as conventional karyotyping does.

In particular, masked hypodiploidy—where a hypodiploid clone undergoes endoreduplication (e.g., 30 chromosomes duplicating to appear as 60)—may go unrecognized by OGM. The method registers two copies of each genomic region but lacks the ability to distinguish between homologous chromosomes and duplicated segments. This may lead to misclassification of a genomically adverse subtype as pseudodiploid or even cytogenetically normal, potentially impacting both prognostic assessment and therapeutic decision-making [4].

### 4.4. Limitations of OGM in Detecting IGH::CRLF2 Fusions in PAR1 Regions

Certain gene fusions, such as *IGH::CRLF2*, located within the pseudoautosomal region 1 (PAR1)**,** are not reliably detected by Optical Genome Mapping (OGM). This limitation is primarily attributable to the cryptic nature of the breakpoints, which often reside in structurally complex, highly repetitive, and poorly mappable genomic regions.

In particular, OGM exhibits suboptimal resolution in PAR loci, where inefficient alignment of ultra-high-molecular-weight DNA molecules compromises detection sensitivity. Consequently, confirmation of fusions involving IGH and CRLF2 typically requires orthogonal, high-sensitivity approaches such as deep whole-genome sequencing (WGS) or targeted methods including PCR and Sanger sequencing.

This diagnostic blind spot carries significant clinical implications, as *IGH::CRLF2* rearrangements are enriched in Ph-like B-cell acute lymphoblastic leukemia (Ph-like B-ALL) and have been associated with high-risk disease features and poor clinical outcomes [6].

### 4.5. Limited Coverage in Repetitive or Telomeric Regions

OGM may exhibit limited coverage in genomic regions characterized by high repetitiveness, telomeric content, or significant sequence homology—factors that impair accurate mapping of ultra-long DNA molecules. These limitations become particularly relevant in the context of complex rearrangements occurring in such regions, including the *IGH::DUX4* fusion, which is located in the telomeric portion of chromosome 4q, near highly repetitive sequences.

DUX4 rearrangements, including *IGH::DUX4*, are associated with a favorable prognosis in B-cell acute lymphoblastic leukemia (B-ALL). However, detection of these events by OGM may be suboptimal, necessitating the use of complementary high-sensitivity methods such as RNA sequencing, whole-genome sequencing (WGS), or targeted PCR to ensure accurate diagnostic identification [26].

### 4.6. Necessity for Complementary Testing to Enable Timely Therapeutic Intervention

While Optical Genome Mapping (OGM) offers a comprehensive approach for identifying complex genomic rearrangements and gene fusions, its longer turnaround time limits its utility for urgent clinical decision-making compared to rapid, targeted assays such as fluorescence in situ hybridization (FISH). 

The *BCR::ABL1* FISH assay remains a gold standard—highly sensitive, rapid, and extensively validated—critical for the swift detection of the *BCR::ABL1* fusion, particularly in diseases where this aberration constitutes the primary pathogenic driver, including chronic myeloid leukemia (CML) and select acute lymphoblastic leukemia (ALL) subtypes.

In these contexts, early and accurate molecular diagnosis is paramount to promptly initiate tyrosine kinase inhibitor (TKI) therapy, which dramatically improves patient outcomes. Accordingly, OGM is best positioned as a complementary, second-tier diagnostic tool, useful for confirming or further resolving the *BCR::ABL1* fusion and for uncovering additional structural variants undetectable by conventional FISH or RT-PCR methodologies [26].

## 5. Discussion

Optical Genome Mapping (OGM) is emerging as a cutting-edge and promising technology for the genomic characterization of acute lymphoblastic leukemia (ALL), offering an integrated, high-resolution alternative to traditional diagnostic modalities. One of its most compelling advantages is the ability to detect multiple classes of genomic alterations—namely structural variants (SVs), copy-number variations (CNVs), deletions, translocations, and gene fusions—in a single assay. This consolidates what has historically required a combination of cytogenetics, FISH, array-CGH, and PCR-based methods into a streamlined workflow [27]. This capability is particularly valuable in ALL, where alterations such as *IKZF1* deletions confer adverse prognostic implications and drive the adoption of more intensive therapeutic strategies. Similarly, OGM’s broad coverage and resolution facilitate the detection of complex gene fusions, including those involving *KMT2A*, which critically shape phenotype and treatment response. Additionally, the technology’s independence from cell culture—mandated by conventional cytogenetic methods—not only accelerates diagnostic timelines but also mitigates artifacts inherent in in vitro culture. Moreover, the ability to analyze ultra-high-molecular-weight DNA molecules opens a window into cryptic or structurally complex genomic regions, enabling the discovery of previously undetectable aberrations by short-read sequencing technologies [28].

Nonetheless, intrinsic limitations indicate that OGM cannot yet serve as a standalone diagnostic tool in ALL. It fails to reliably detect balanced rearrangements located within highly repetitive and structurally complex genomic areas—such as centromeres and short arms of acrocentric chromosomes—limiting its capacity to recognize Robertsonian translocations like rob(15;21), which are associated with i*AMP21*–ALL. The inability to detect such events undermines clinical stratification unless complemented by methods such as karyotyping or FISH. OGM also demonstrates shortcomings in interpreting complex ploidy states. For example, in cases of masked hypodiploidy due to endoreduplication, OGM may register a seemingly normal diploid status by reporting two copies per region, obscuring an adverse chromosomal status with significant prognostic implications. Moreover, OGM does not resolve breakpoint sequences in cryptic or highly repetitive regions, such as *IGH::CRLF2* in PAR1 or *IGH::DUX4* within distal 4q telomeric areas. These fusions have important clinical relevance—e.g., *IGH::CRLF2* in Ph-like B-ALL, or DUX4 fusions associated with favorable prognosis—but require confirmation via high-resolution sequencing approaches such as high-depth WGS, RNA-seq, targeted PCR, or Sanger sequencing to map breakpoints accurately [29].

Practically, OGM is best utilized as a complementary, rather than a substitutive, technology in the diagnostic algorithm for ALL. When integrated with NGS, OGM provides a comprehensive molecular portrait: OGM excels at detecting large structural variants and CNVs, while NGS delivers high sensitivity for point mutations and smaller sequence-level changes. This multimodal diagnostic strategy is essential for achieving a thorough, clinically meaningful molecular characterization of ALL, underpinning accurate risk stratification and personalized treatment planning [30].

## 6. Conclusions

Optical Genome Mapping represents a frontier in the molecular diagnosis of acute lymphoblastic leukemia, furnishing an integrated, high-resolution genomic perspective capable of simplifying and expediting diagnostic workflows. Its capacity to simultaneously detect a wide array of structural and copy-number alterations, including complex or cryptic events, marks a significant leap beyond traditional methodologies, thereby enhancing prognostic accuracy and informing more targeted therapeutic approaches. Nonetheless, the current limitations in detecting alterations in highly repetitive genomic regions, resolving precise breakpoints, and correctly interpreting complex ploidy require that OGM be integrated with established cytogenetic and sequencing-based techniques—such as NGS—to deliver comprehensive and clinically actionable results. Real-world experience suggests that pairing OGM with conventional and NGS platforms improves diagnostic precision, shortens turnaround times, and deepens biological insight, ultimately benefiting patient management. Looking forward, advancements in sequencing technologies and enhancements in analytical workflows may further elevate OGM toward becoming the diagnostic cornerstone in ALL and broader hematologic malignancies, promoting more refined therapeutic personalization and improved clinical outcomes.

## Figures and Tables

**Table 1:** Advantages of OGM in ALL.

Advantage	Description
Comprehensive Genomic Profiling	Enables simultaneous detection of structural variants (SVs), copy number variations (CNVs), deletions, translocations, and gene fusions within a single assay, streamlining diagnostics.
No Requirement for Cell Culture or Amplification	Requires minimal cell input without the need for mitotic cell culture or PCR amplification, allowing analysis of cryopreserved or fixed samples and reducing amplification biases.
High Sensitivity and Resolution	Provides superior breakpoint precision and detects cryptic or complex rearangements missed by conventional cytogenetics and FISH, improving risk assessment and prognostic accuracy.
Detection of Novel and Cryptic Rearrangements	Identifies previously unrecognized balanced and unbalanced genomic rearrangements with potential diagnostic, prognostic, and therapeutic relevance.
Reduced Diagnostic Turnaround and Complexity	Consolidates multiple conventional tests into a single platform, reducing turnaround times to 4–7 days and lowering per-sample costs by up to 50%, enhancing clinical workflow efficiency.
Complementary to Next-Generation Sequencing (NGS)	Complements NGS by resolving large-scale structural variants and CNVs with high resolution, while NGS detects small mutations, together providing a comprehensive genomic landscape.

**Table 2:** Gene Fusions and Translocations Identified in ALL via OGM.

Genomic Event	Description and Clinical Relevance
Precise identification of the *ETV6::ABL1* fusion	Rare but clinically relevant subtype associated with poor prognosis; sensitive to tyrosine kinase inhibitors (TKIs). OGM enables high-resolution detection even when the fusion is cryptic and structurally complex.
Novel *WDFY2::ARID2* gene fusion	t(12;13) translocation possibly causing *ARID2* haploinsufficiency, a key regulator of hematopoietic stem cell differentiation. Potential biomarker for immune checkpoint blockade therapy response.
*FLI1::EWSR1* fusion	Rare, non-canonical event in ALL linked to adverse clinical outcomes and resistance to standard treatments. May disrupt cell cycle regulation and promote an aggressive disease phenotype.
Novel *TMEM272::KDM4B* fusion (t(13;19))	Fusion with unclear oncogenic potential. *KDM4B* is frequently overexpressed in solid tumors and associated with genomic instability. Its role in ALL requires further investigation.
Novel *LMNB1::PPP2R2B* fusion	Involves *LMNB1* (nuclear lamina component) and *PPP2R2B* (PP2A regulatory subunit). Potential role in leukemogenesis; often co-occurs with *ETV6::RUNX1* fusion. Further functional studies needed to clarify therapeutic implications.
*JAK2::NPAT* fusion	Constitutive activation of *JAK2* kinase driving leukemogenesis; targetable by selective JAK inhibitors like ruxolitinib. Relevant in a molecularly defined ALL subset.
Novel *OSBPL3::NRIP1* fusion	Involves proteins linked to bone homeostasis and transcriptional regulation. Rare event with limited clinical data; further research needed to determine biological and therapeutic significance.
Complex translocation t(5;12;21)	Pathogenic role not fully understood; isolated t(5;12) reported in chronic eosinophilic leukemia responsive to dasatinib. Highlights potential for targetable clonal evolution in ALL; longitudinal molecular monitoring recommended.
Novel *RUNX1::SPG7* fusion	*RUNX1* is a well-known leukemia oncogenic fusion partner; *SPG7*, involved in mitochondrial function, not previously linked to oncogenic fusions. Clinical and functional significance currently unclear; requires further investigation.

**Table 3:** Limitations of Optical Genome Mapping (OGM) in Acute Lymphoblastic Leukemia (ALL).

Limitation	Description	Impact on ALL Diagnosis	Complementary Methods Needed
Balanced rearrangements in repetitive regions	Difficulty detecting balanced chromosomal rearrangements in centromeres and acrocentric chromosome short arms.	Missed Robertsonian translocations (e.g., rob(15;21))	Conventional karyotyping, FISH
Low variant allele frequency (subclonalvariants)	Reduced sensitivity for structural variants in minor subclones with low allele frequency.	Missed clinically relevant subclones	RT-PCR, NGS
Complex ploidy states	Difficulty distinguishing hypodiploidy, masked hypodiploidy, euploidy, and hyperdiploidy due to lack of copy number quantification.	Misclassificatio of risk subtype	Conventional karyotyping
*IGH::CRLF2* fusions in PAR1 region	Limited detection in pseudoautosomal regions due to cryptic breakpoints and repetitive sequences.	Missed high-risk fusions in Ph-like ALL	PCR, Sanger sequencing, deep WGS
Coverage in repetitive/telomeric regions	Poor mapping in highly repetitive or telomeric regions.	Suboptimal detection of fusions like *IGH::DUX4*	RNA sequencing, targeted PCR, WGS
Turnaround time for urgent decisions	Longer processing time limits use for rapid clinical decision-making.	Delay in *BCR::ABL1* fusion detection for early TKI therapy	FISH, RT-PCR
